# Gel immersion echoendoscope-guided puncture before radial incision and cutting for complete rectal anastomotic obstruction

**DOI:** 10.1055/a-2106-1744

**Published:** 2023-06-27

**Authors:** Shozo Osera, Takeshi Hisa, Gaku Akiyama, Akiharu Kudo, Takahiro Yamada, Hideki Fukushima, Akihisa Tomori

**Affiliations:** 1 Department of Gastroenterology, Saku Central Hospital Advanced Care Center, Nagano, Japan; 2 Department of Colorectal Surgery, Saku Central Hospital Advanced Care Center, Nagano, Japan

Benign anastomotic complete obstruction rarely occurs after lower rectal cancer surgery 1 . The radial incision and cutting (RIC) method has been reported for complete rectal anastomotic obstruction 2 ; however, it is important to penetrate the distal and proximal sides of the intestinal tract safely and accurately before RIC. A forward-viewing echoendoscope is useful for recanalizing postoperative biliary anastomotic atresia in endosonography-guided biliary drainage because it allows a more vertical approach and shortens the puncture distance 3 4 . Furthermore, gel-immersion techniques have been reported for endoscopic procedures 5 .

A 59-year-old man underwent intersphincteric resection and temporary ileostomy for lower rectal cancer. Ileostomy closure was scheduled for 12 months after the surgery. Endoscopic imaging revealed complete rectal anastomotic obstruction 2 cm from the anal verge ( Fig. 1 ). We attempted an endoscopic intervention to avoid a surgical procedure.

**Fig. 1:**
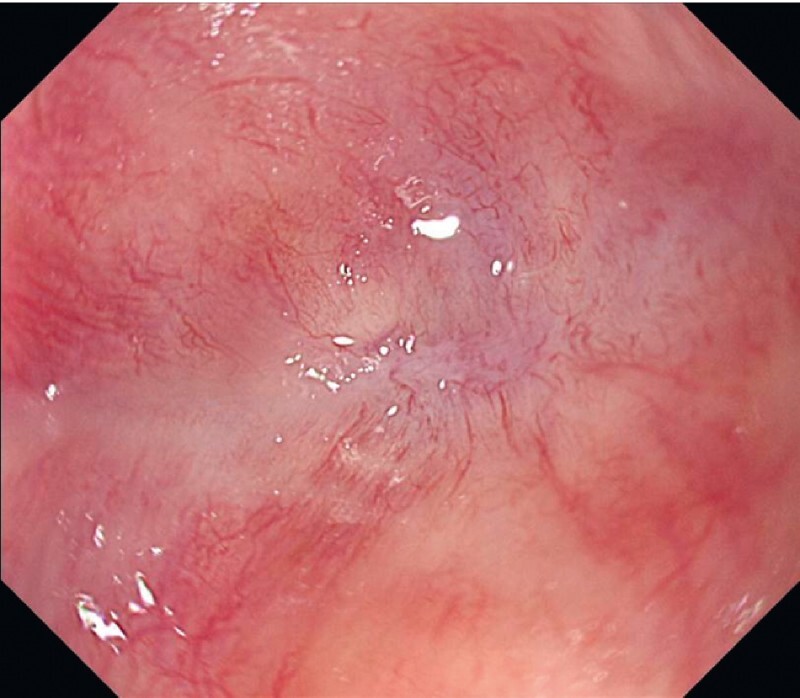
The site of the lower rectal anastomosis at 2 cm from the anal verge.

A forward-viewing convex echoendoscope (TGF-UCT260J; Olympus Medical Systems, Tokyo, Japan) and immersed gel (VISCOCLEAR; Otsuka Pharmaceutical Factory, Tokushima, Japan) was inserted through the anus. Gel immersion provided a clear endoscopic ultrasound view and helped identify the puncture line ( Fig. 2 ). We inserted a 19-gauge needle (EZ Shot 3 plus; Olympus Medical Systems) toward the proximal intestinal tract ( Video 1 ). We confirmed patency of the proximal lumen using contrast enhancement and placed a 0.025-inch guidewire (VisiGlide 2; Olympus Medical Systems). Dilation was performed using a 4-mm biliary dilation balloon catheter (REN; Kaneka Medix Corp., Osaka, Japan) until the notch disappeared ( Fig. 3 ), and performed RIC using an ITknife nano (KD-611L; Olympus Medical Systems) ( Fig. 4 ). After the procedure, an endoscope with a 9.9 mm diameter could penetrate the anastomotic site ( Fig. 5 ).

**Fig. 2:**
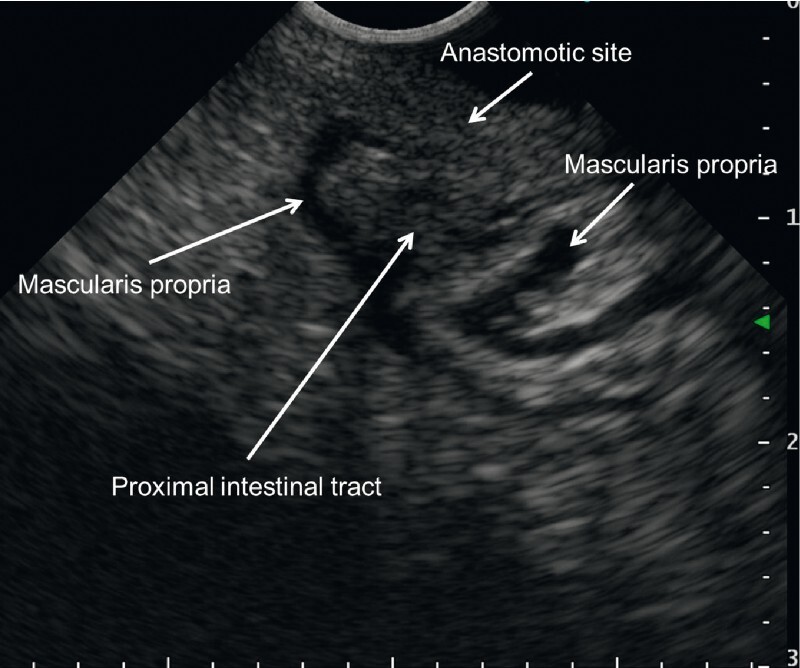
Gel immersion endoscopic ultrasound view clearly showed the proximal intestinal tract.

**Video 1** Gel immersion forward-viewing echoendoscope-guided puncture before radial incision and cutting with endoscopic balloon dilation for complete rectal anastomotic obstruction.


**Fig. 3:**
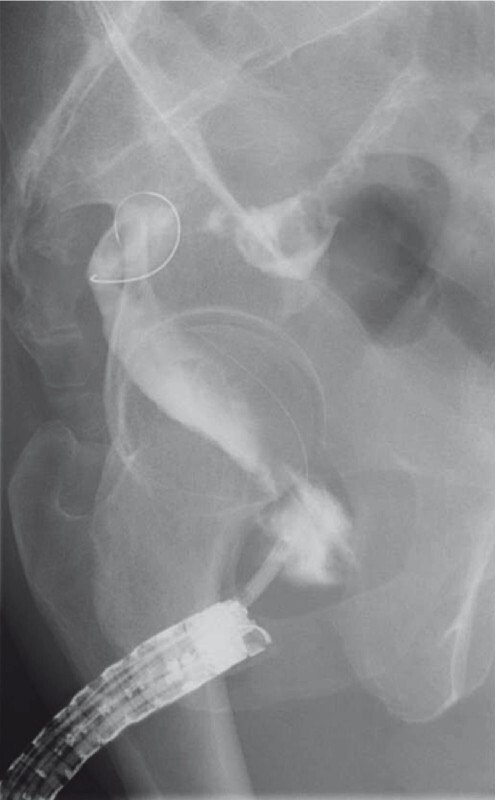
Fluoroscopic view of endoscopic dilation using a 4-mm biliary dilation balloon catheter until the notch disappeared.

**Fig. 4:**
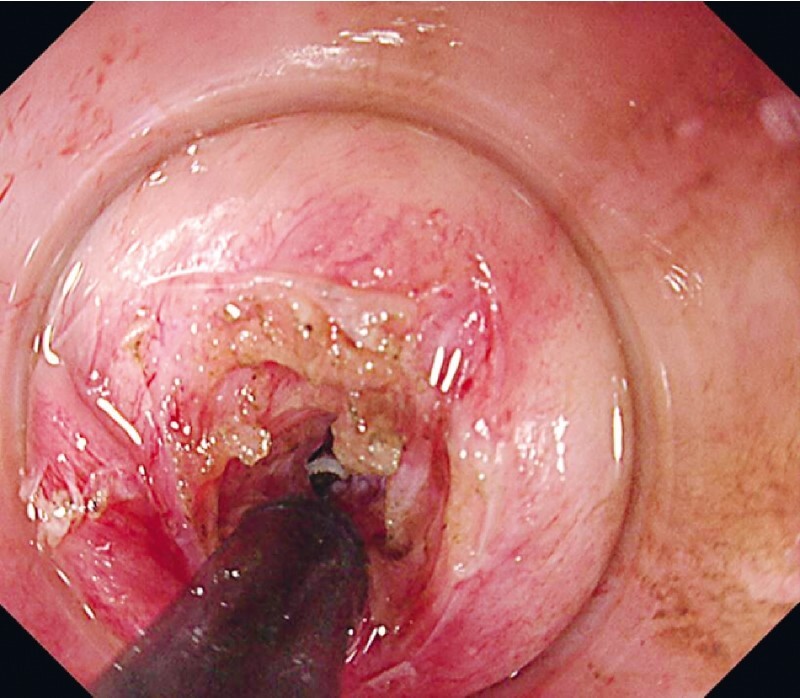
The radial incision and cutting method using an ITknife nano (KD-611L; Olympus Medical Systems, Tokyo, Japan).

**Fig. 5:**
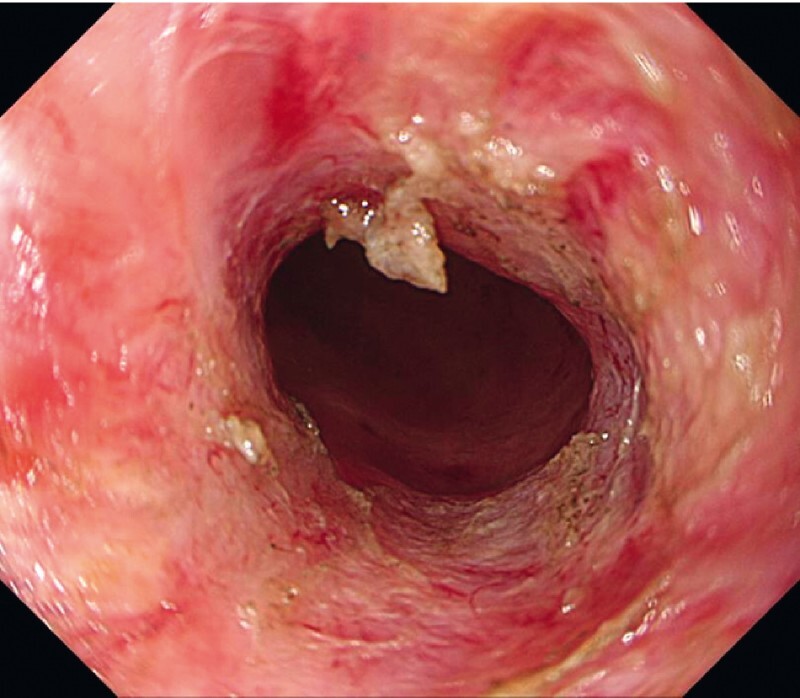
After the radial incision and cutting procedure, an endoscope with a 9.9 mm diameter could pass through the anastomotic site.

Forward-viewing echoendoscope-guided puncture using gel immersion before RIC with endoscopic balloon dilation is a safe and effective procedure for resolving anastomotic obstructions after lower rectal surgery.

Endoscopy_UCTN_Code_TTT_1AS_2AZ

## Notes

Endoscopy E-Videos

https://eref.thieme.de/e-videos
*E-Videos* is an open access online section of the journal *Endoscopy* , reporting on interesting cases and new techniques in gastroenterological endoscopy. All papers include a high-quality video and are published with a Creative Commons CC-BY license. Endoscopy E-Videos qualify for HINARI discounts and waivers and eligibility is automatically checked during the submission process. We grant 100% waivers to articles whose corresponding authors are based in Group A countries and 50% waivers to those who are based in Group B countries as classified by Research4Life (see: https://www.research4life.org/access/eligibility/ ). This section has its own submission website at https://mc.manuscriptcentral.com/e-videos
